# Patients who discontinued statin treatment: a protocol for cohort study using primary care data

**DOI:** 10.1136/bmjopen-2015-008701

**Published:** 2015-10-22

**Authors:** Yana Vinogradova, Carol Coupland, Peter Brindle, Julia Hippisley-Cox

**Affiliations:** 1 Division of Primary Care, University of Nottingham, Nottingham, UK; 2 Bristol Clinical Commissioning Group, Bristol, UK

**Keywords:** statins, discontinuation, cardiovascular disease

## Abstract

**Introduction:**

Risk thresholds for using statins to prevent cardiovascular disease (CVD) have recently been lowered, so an increasing number of patients are now prescribed these drugs. Although the safety of long-term statin use has been generally established, concerns about the balance of risks and benefits of statins still exist for some medical professionals and patients, and issues concerning their side effects are occasionally widely publicised. This study will report the rates of stopping for statins and also identify any patient groups more likely to stop using statins, so possibly increasing their risk of cardiovascular events.

**Methods and analysis:**

A prospective open cohort study between 1 January 2002 and 30 September 2014 will be based on the general population of people prescribed statins, using records from UK general practices contributing to the Clinical Practice Research Database (CPRD). Participants aged 25–84 years will enter the cohort on the date of their first prescription for a statin and leave on the earliest date of: a cardiovascular event; death; leaving the practice; the last practice upload date or the study end date. If there are no prescriptions within 90 days after the expected finishing date of a prescription, a patient will be defined as a stopper with the discontinuation outcome date as the expected finishing date. Rates of statin discontinuation will be calculated by calendar year, type and dose of statin, age, and morbidities. Cox proportional regression analyses will be run to identify the most important factors associated with discontinuation. Analyses will be run separately for patients without CVD (primary prevention) and with diagnosed CVD (secondary prevention).

**Ethics and dissemination:**

The protocol has been reviewed and approved by Independent Scientific Advisory Committee for MHRA Database Research. The results will be published in a peer-reviewed journal.

**Strengths and limitations of this study**

- Large size and substantial statistical power.
- Representative study population.
- Uncertainty regarding adherence to statin treatment and exact date of the statin discontinuation.
- Possible underestimation of statin use due to over-the-counter use.
- Lack of information on some factors that might affect patients’ decisions to stop statins.

## Introduction

Cardiovascular disease (CVD), which includes heart attacks and strokes, has a major impact on the lives of affected individuals and treatment costs are a significant burden for health services.^1^ A high level of low-density lipoprotein (LDL) cholesterol is a significant risk factor for CVD^2^ and statins are frequently prescribed as a preventive measure to reduce cholesterol levels. In the UK in 2006, the National Institute for Health and Care Excellence (NICE) recommended statins for primary prevention in patients with a 10-year risk of CVD of 20% or more.^3^ Subsequently, the Cholesterol Treatment Trialists meta-analysis (2012),^4^ which was based on 27 trials of outcomes for statin users versus controls, demonstrated in lower risk patients a risk reduction for major vascular events of 21% per 1.0 mmol/L from LDL cholesterol level reduction, and in 2014, NICE decreased risk threshold for recommending statin prescribing to a 10-year risk of 10%.^5^ In the USA, the thresholds for using statins have also recently been lowered,^6^ so many more patients are eligible to receive prescriptions for these drugs.

The benefits of such preventive therapy are, however, dependent on the level of adherence of patients with their prescribed regime and discontinuation is an extreme form (zero adherence) of non-adherence.^7^ In general, non-adherence to prescribed medication is associated with adverse outcomes and higher costs of care.^8^ Discontinuation of statins can cause changes in platelet activity or inflammation, impair vascular homoeostasis or lead to endothelial dysfunction and may, therefore, independent of changes of cholesterol levels, increase risk of cardiovascular events.^9^ A number of studies in patients with acute cardiovascular conditions have demonstrated problems associated with statin withdrawal. In one, hospitalised patients who discontinued statins were found to have a three times higher rate of myocardial infarction (HR 2.93, 95% CI 1.64 to 6.27) than patients who continued to receive statins.^10^ Other studies have demonstrated that stopping statins after an acute cardiovascular event increased mortality risk or worsened the outcome.^11^ ^12^ One randomised controlled trial in patients with stable cardiac conditions found no significant differences in rates of cardiac events or deaths from heart disease associated with statin discontinuation, but the number of actual events was too low to allow for the effect of statin non-adherence—24 in the prior 6-week wash-out period compared with 31 in the 8-week therapy period.^13^

However, while statin therapy is associated with decreased mortality and fewer complications related to atherosclerosis for patients already diagnosed with CVD, this has not been established for patients without CVD. Statins also have side effects and a number of studies have demonstrated these,^14^ which include increased risks for diabetes^15^ ^16^ and myopathy.^17^ ^18^ With respect to their effectiveness in patients without CVD, a meta-analysis of 11 randomised controlled trials reported no significant associations between statin use and mortality risk for such patients (risk ratio 0.91, 95% CI 0.83 to 1.01).^19^ A later meta-analysis, investigating the effect of cholesterol-lowering therapy on all-cause mortality, did show a significantly reduced risk for patients without CVD (risk ratio 0.91, 95% CI 0.88 to 0.93).^4^ However, as highlighted in a subsequent secondary analysis, this reduced mortality was not demonstrated for the subgroup with low cardiovascular risk (0.95, 0.86 to 1.04 for CVD risk <10%).^20^ Moreover, the original finding might simply have reflected a healthy-user bias because a study investigating the risk of accidents associated with adherence to statins^21^ has shown adherent patients to be less likely to be involved in accidents (HR 0.85, 95% CI 0.83 to 0.87) and more likely to use screening services (1.17, 1.15 to 1.20).

While the safety of long-term statin use has, therefore, been supported by systematic reviews,^4^ ^22^ issues about their side effects, effectiveness and the balance of risks and benefits, especially for people at low risk of CVD, have occasionally been widely publicised.^20^ ^23^ So concerns do still exist among some medical professionals and their patients, and statin non-adherence and discontinuation seem likely to rise as more low-risk patients are included, who have no symptoms and might regard such preventive medications as unimportant or risky.^24^ ^25^

Non-adherence to drug treatments is, however, a many-layered issue, which can be related to a poor healthcare system, the type of condition, patient characteristics, the complexity or side effects of the therapy, or specific socioeconomic issues for a treatment group.^8^ For statins in a UK study environment, issues to do with the health system or cost of treatment are unlikely to be significant. Taking statins by themselves is also not a complex therapy, although issues arising from polypharmacy or other patient characteristics might well cause discontinuation. Rates of non-adherence to statins in cardiovascular patients has, however, been shown to be 26%,^26^ while a recent American study has reported that more than 50% of statin users stopped treatment at least temporarily.^27^

The proportion of patients who discontinue statins and their reasons for doing so are not, however, clear. Discrepancies between randomised controlled trials and observational studies have been shown in a meta-analysis,^28^ which reported for randomised trials a 90.3% (95% CI 89.8% to 90.8%) adherence at 1-year follow-up against 49% (95% CI 48.9% to 49.2%) for observational studies. This high adherence found in randomised controlled trials is clearly related to the enrolling of selected populations and provision of a form of monitoring for the included patients. Observational studies provide a much closer reflection of the real world, but the very high levels of discontinuations suggest that many may not be due to statin-related events, and this has led to a number of studies investigating patterns of discontinuation and the causes.

Of these, all seem to have concentrated on patients with already diagnosed CVD, and most were conducted either before the most recent CVD guidelines were issued or UK-derived CVD risk estimation was available. One study, based on Clinical Practice Research Database (CPRD) data, which showed an association between discontinuation and higher total mortality following acute myocardial infarction,^29^ has also provided information about prevalence of comorbidities with respect to different patterns of statin use. Another CPRD study has investigated the factors associated with discontinuation,^30^ but this study was conducted in 2002. This assessed discontinuation risks in a mixed group of patients, but having only 20% of the cohort without diagnosed CVD and following up patients for only 1 year. It has also been noted that further research is needed particularly for patients with low cardiovascular risk.^31^

While there have been observational studies examining the pattern of uptake of statins^32–34^ and of unintended effects from statin use,^35–37^ relatively few studies have examined the discontinuation of statins. We have, therefore, designed a study that will follow statin users in the UK over time, report the rates of stopping for statins with the discontinuation outcome based on prescription data, and look at whether these have changed with time or differ across statin types. The study will also identify any patient groups more likely to stop statins and so possibly increase their risk of CVD.

## Methods and analysis

### Data source

We will use data from 664 UK general practices contributing to the CPRD (http://www.cprd.com). The practices are spread across the UK, and their registered patients have been shown to be representative of the general population.^38^ Data are routinely collected and the recorded information includes demographics, patient characteristics, clinical diagnoses, symptoms and prescribed medications. The database has been validated using other sources of information in the UK and has been used for similar studies for statin discontinuation.^29^ ^30^

### Sample selection

This will be a prospective open cohort study of new statin users identified during the study period between 1 January 2002 and 30 September 2013. Incident cases who discontinued statin therapy will be identified during follow-up. Patients will enter the cohort on the date of their first recorded statin prescription within the study inclusion period. Patients will be followed up until the earliest of: death; deregistration with the practice; last upload of computerised data; 1 year from the date the study inclusion period ended. Patients will be subdivided into two groups evaluated on the date when they started taking statins: those without CVD records before the entry date (taking statins for primary prevention); those with CVD diagnosed at or before entering the study (taking statins for secondary prevention). Patients without CVD at the baseline date will be followed up until the time of a CVD diagnosis if it occurs before the study exit date.

We expect the rate of discontinuation to be lower in the secondary prevention group, and this subdivision will make our results more easily comparable with existing studies.

### Inclusion criteria

Eligible patients will be aged 25–84 years at the study entry, have at least two prescriptions for a statin in the study period and have at least 1 year of records prior to entry—calculated from the later date of: the date of registering with a general practice or the earliest date of upload of computerised practice data—and at least 1 year of follow-up—calculated from their first statin prescription in the study period. Patients with one or more prescriptions for statins in the 12 months prior to cohort entry will be excluded. Cerivastatin will not be included in the study because it was withdrawn from the market in August 2001, but patients with prescriptions for it in the 12 months prior to cohort entry will be excluded.

### Exposure

A number of statin users will have only one prescription before stopping. Because they did not receive a repeat prescription, it cannot be certain that these patients actually ever took statins. So, for the main analysis, having excluded patients with one prescription, we will run a sensitivity analysis including all patients with one or more prescriptions. We will report on the percentage of patients that had only one prescription and describe characteristics of this group.

The following statins will be included into the analysis: atorvastatin, simvastatin, fluvastatin, pravastatin and rosuvastatin. As the statin most commonly prescribed in the overall study period, simvastatin—used by around 70% of patients—will be used as the reference drug.

### Outcome

The outcome will be defined as a discontinuation of statin use if there are no prescriptions within the 90 days following the expected finishing date of a prescription. The first such event will be considered as an outcome, and further prescriptions will not be used in the analysis of discontinuation. A 90-day exposure window has been used in other CPRD studies^29^ ^30^ because it is the longest prescription length that can be issued in the UK. The date of the outcome will be defined as the expected finishing date of the prescription.

### Covariates

To describe the cohort and explore factors associated with the risk of discontinuation, we will consider the following covariates in six major groups. First, demographics will include age (<45; 45–54; 55–64; 65–74; 75–84 years), sex, ethnicity (Caucasian or not recorded, Indian, Pakistani, Bangladeshi, other Asian, Black African, Black Caribbean, Chinese, other ethnic group) and socioeconomic status as measured by the Townsend deprivation score in quintiles. Second, clinical values will include smoking status (non-smoker; ex-smoker; light smoker: <10 cigarettes/day; moderate smoker: 10–19 cigarettes/day; heavy smoker: 20+cigarettes/day; not recorded), body mass index (BMI) in kg/m^2^ (<25; 25–29.9; 30–34.9; 35+, not recorded), systolic blood pressure and cholesterol/high-density lipoprotein (HDL) ratio. Third, chronic diseases will include rheumatoid arthritis, chronic renal disease, liver disease, atrial fibrillation, treated hypertension, cancer, heart failure, diabetes and dementia. Fourth, genetic characteristics will include family history of premature coronary heart disease and diagnosis of familial hypercholesterolaemia. Fifth, other medications will include use of aspirin, use of anticoagulants and—as a proxy for comorbid conditions—the number of different types of medicines having systemic effect and associated with British National Formulary^39^ categories (in each case, at least one prescription in the last year before the entry date). Finally, only for patients in the primary prevention group, their 10-year cardiovascular risk as calculated by the QRISK2–2014 score (banded <5%; 5–9%; 10–14%; 15–19%; 20–24%; 25%+).

For clinical values, the most recently recorded value prior to or at the cohort entry will be used. Chronic diseases will be considered if they were recorded at or before the baseline. For the main analysis, we will use practice-based Townsend deprivation scores as these are available for the whole cohort. We will run an additional analysis on the subset of practices that have consented to link their data to Townsend scores at patient level.

### Statistical analysis

All analyses will be performed separately for the subgroups with and without a diagnosis of CVD at the study entry date.

The numbers of incident users who stop statins will be reported and the rate of discontinuation will be assessed by dividing the number of incident stoppers by the number of person-years. The rates will be calculated overall and by calendar year, ethnic group, deprivation quintile, initial statin type, and baseline risk of CVD (for primary prevention group). We shall also calculate the percentages of patients who switch between different statins during the study period, considering a patient as a switcher if their type of statin (ingredient) changed during their follow-up. For patients in the primary prevention group who were diagnosed with CVD after entering the study, we shall report the percentages of patients who continued using statins after the diagnosis.

We shall report general descriptive statistics such as average duration of statin therapy before discontinuation, proportions of patients who had their last statin prescription within 6 months, 6 months to 1 year, 1–5 years or more than 5 years after their first prescription, and graphically summarise the data using Kaplan-Meier curves. As we expect different rates of discontinuation for short-term (less than 12 months between the first and the last prescription) and long-term (more than 12 months between the first and the last prescriptions) users, we shall calculate rates by term of use.

For those patients who discontinued statins and for those who did not, summary statistics will be calculated for the baseline covariates. We shall report frequencies and proportions for categorical data, and for continuous data, suggested bands as well as means (SD), medians and ranges. We shall also report the type of statin, the potency, calendar year for the baseline, cumulative duration of exposure before leaving the cohort (defined as the time interval between the first prescription and expected end date for the last prescription), and whether the patient had switched statin type during the study period. We shall calculate the survival-time function, report the descriptive statistics such as median duration, plot Kaplan-Meier curves for the covariates and compare the survivor functions for the covariates using the log-rank test.

To explore the risks of statin discontinuation associated with the covariates, a Cox regression analysis will be undertaken. The model will include age, sex, ethnic group, deprivation quintile, calendar year at the entry to the cohort, baseline CVD risk and the type of statin prescribed at the baseline. Practices might differ in their prescription patterns, so we will account for clustering by practice. We shall calculate the adjusted HRs for baseline characteristics listed above: clinical values; chronic diseases; family history; use of other medications.

The proportional hazards assumption will be checked by using a log-log plot of survival for each variable. Variables which violate the assumption will be included into the model as a stratification factor. If clinically similar variables or categories (for smoking and ethnicity) are associated with similar risks, they will be combined. For continuous variables, recorded values will be used. For non-linear risk associations, fractional polynomials will be used if appropriate.^40^

As BMI, smoking status, systolic blood pressure and cholesterol/HDL ratio may be important patient characteristics but have non-negligible numbers of missing data, multiple imputation will be used to replace missing values.^41^ Five imputed data sets will be created, using multiple chained equations, and Rubin's rules will be used to combine effect estimates and SEs to allow for uncertainty due to imputing missing values. Calendar year; statin stopper status; log of survival time; age; years of records; potentially important characteristics; and type, dose and cumulative exposure to statins will be included in the imputation model.^42^

The main analyses will be run using the multiply imputed data. The least complete data are expected to be for cholesterol/HDL ratio, but information about this test is expected to be important for patient adherence. To ensure the robustness of our findings, we shall also run a sensitivity analysis including only patients with complete data.

### Additional analyses

Patients might discontinue statins within the first year or after a longer period. The characteristics of these patients might differ, so we shall rerun the main analysis twice, in the first censoring all patients who did not discontinue before the end of year one, and in the second removing all patients who did discontinue before the end of year 1.

Some statin users have discontinuous use, with one or more restarts against a background of fairly continual usage. We shall define a subcohort in the study period for patients who discontinued statins (the outcome date in the main analysis will be their new entry date) and follow them until they have a new statin prescription or they are censored as described in the Study design section. We shall describe how many people restarted statin therapy within the first 6 months, 7–12 months and 12 or more months after their new entry dates using Kaplan-Meier estimates. We shall report descriptive statistics for the covariates listed above both for stoppers who restarted and those who did not.

### Sensitivity analyses

Apart from the two sensitivity analyses already mentioned relating to exposure and missing values, two others will be included. One will be to facilitate comparison with the results of the Zhang *et al*^27^ study for long-term statin discontinuation. In this, we will define an outcome as an absence of any statin prescriptions within the 12 months (365 days) following the expected finishing date of a prescription. As before, the expected last date will also be the date of the outcome. Because of the longer period required for an outcome, for this analysis, we will remove patients with less than 2 years of follow-up records. The other will be run to investigate the influence of possible changes in baseline variables over the full study period, by rerunning the analysis with the follow-up period restricted to 5 years.

A 1% level of statistical significance will be used to allow for multiple comparisons. Stata V.13 will be used for all the analyses.

### Sample size calculation

All eligible patients will be used in the analyses, allowing investigation of different types of statins and facilitating identification of relevant patient characteristics associated with discontinuation. This approach is particularly important for the smaller group of patients already diagnosed with CVD at baseline.

About one million patients with at least one prescription of statin within the study period are available from the CPRD. Of these, just over one-third had a statin prescription in the past 12 months before the study entry or insufficient data for follow-up. Excluding these will leave about 600 000 patients available for analysis, one-quarter with CVD at the baseline.

For individual drug exposure, 5362 observations will be required to detect a HR of 0.9 or 1.1 at a significance level of 1% with 90% power between simvastatin and other statins. For common drugs such as atorvastatin and rarer drugs such as pravastatin (around 5% of exposure) we will have enough observations for all these drug types for both CVD and non-CVD groups. For rare drugs, such as rosuvastatin (less than 2% of exposure) and fluvastatin (less than 1% exposure), 1196 patients will be required to detect a clinically important HR of 0.8 at a significance level of 1% with 90% of power. We will have enough observations for both the CVD and non-CVD group for rosuvastatin, but only for the non-CVD group for fluvastatin.

## Discussion

This is an observational study based on routinely collected data from a large primary care research database and will have the strengths and limitations common to such studies. The strengths include the representativeness of the study population, large size of the sample and substantial statistical power. Information about discontinuation rates is scarce, and no previous study has described discontinuation rates in the general population separately for primary and secondary prevention. Our paper will also report on a significantly longer follow-up time than previous studies.^27^ ^28^

The limitations of the study will include potential overestimation of statin use. While we have precise dates on which prescriptions were issued, we do not have detailed information about whether patients took their pills, when they started and stopped taking the pills, or whether they followed the prescribed regime, although it is likely that most patients with more than one prescription were genuine users.^43^ There is also a possibility of underestimation of statin use because simvastatin has been available over-the-counter since 2004, and some patients might have obtained it without returning to the general practitioner for repeat prescriptions. These would appear as false stoppers, but a Health Survey for England has shown the proportion of patients using over-the-counter sources (restricted by license to individuals with a 10–15% 10-year CVD risk) to be very low at 0.2%.^44^ Another limitation is the lack of information on reasons for statin discontinuation, such as side effects or no further need for the drug. Although some of this information may be recorded, it is too sparse to be used in the analysis.

Not all of the possible factors associated with statin discontinuation, which have been reported by other studies, will be available for our analysis. Some of them might have proxies in the form of a similar association, for example, the social deprivation category in our study for levels of education or income. Other factors, such as the quality of communication between patient and doctor or the patient's understanding of information on statins^45^ are not recorded in primary care databases and would be infeasible to include in a large study of this kind.
